# Improvements in Brazed-Joint Properties of Silicon Nitride and Titanium Alloys Using Laser-Induced Microscale Rice Leaf Structures

**DOI:** 10.3390/ma15196750

**Published:** 2022-09-29

**Authors:** Jian-Guo He, Shou-Jun Dai, Yang Zhao, Min Huang, Yang Liu, Jia-Qi Yu, Yu Tan, Lian-Wen Fan, Wen-Qi Ge, Yun-Feng Ma

**Affiliations:** 1 Aerospace Information Research Institute, Chinese Academy of Sciences, Beijing 100094, China; 2 University of Chinese Academy of Sciences, Beijing 100049, China; 3 Key Laboratory of Computational Optical Imaging Technology, Chinese Academy of Sciences, Beijing 100094, China; 4 Beijing Institute of Spacecraft System Engineering, Beijing100094, China; 5 State Key Laboratory of Transient Optics and Photonics, Xi’an Institute of Optics and Precision Mechanics of CAS, Xi’an 710119, China; 6 Technology and Engineering Center for Space Utilization, Chinese Academy of Sciences, Beijing 100094, China

**Keywords:** laser-induced periodic surface structure, roughness, brazing, microstructure, laser welding

## Abstract

Si_3_N_4_ ceramics with a microscale rice leaf structure (MRLS) and titanium alloy were connected via brazing, and the influence of the surface microstructure on the ceramic connection was analyzed. MRLS fabrication is an efficient and high-degree-of-freedom method that can be used to change a material’s surface morphology and wettability. The MRLS was obtained at a laser power of 110 W, with line spacings of 100 and 50 μm. The laser-treated surface included nanoparticles and micro particles, exhibiting a coral-like structure after agglomeration. When the MRLS was used to braze the titanium alloy, no defects were observed at the brazing interface, and the formation was excellent. Throughout the brazed joint, the MRLS remained intact and formed a strong metallurgical bond with the brazing filler metal. A finite element analysis was performed to study the cross-sectional morphology after joint fracture; from the load-time curve, it was found that the MRLS on the surface not only helped improve the mechanical occlusion and brazing area at the interface, but also helped generate compressive stress on the Si_3_N_4_ side. Crack propagation was hindered, thereby increasing the joint strength.

## 1. Introduction

Ceramics are widely used in aerospace, mechanical engineering, communications, electronics, and automobile fields; this is due to their incomparable high-temperature resistance, corrosion resistance, wear resistance, and unique electrical properties. Si_3_N_4_ ceramics exhibit high toughness, flexural strength, and excellent thermal insulation properties; their thermal expansion coefficient is close to that of steel and they are widely used in the field of structural ceramics [1,2,3]. However, the processability of Si_3_N_4_ is poor because of its high hardness and brittle texture. Further, it is difficult to directly process large-scale structural parts, significantly hindering its application. Therefore, the emergence of Si_3_N_4_-joining technology is expected to promote its development. Currently, there are many connection methods, including diffusion, brazing, reaction joining, etc. [4,5,6]. The direct brazing of ceramics involves the addition of active elements to the brazing filler, such that these elements react with the ceramic interface, thereby improving their wettability and producing a better joining effect [7,8].

Laser-induced periodic surface structures (LIPSSs) are important for fabricating surface microstructures [9,10,11,12]. LIPSSs are periodic nanostructures fabricated on the surface of metal materials using a pulsed laser to prepare micro/nano secondary surfaces with hydrophilic structures [13,14]. Chen et al. [15] used a laser to texture Ti_3_SiC_2_ ceramics to improve their wettability. After surface treatment, the wetting angle of the brazing filler on the ceramic was reduced from 59.6° to 25.7°, and the wettability was significantly improved. The brazing filler was uniformly filled into the machining groove, and the interface-forming effect was excellent. The surface microstructure morphology had a direct impact on wettability. With the change in the surface microstructure spacing, the wetting angle fluctuated [16]. Liu et al. [17] found that the Al–Si alloy had poor wettability on the surface of microgrooves and micropits, whereas the Al–Si alloy showed better wettability on the micro/nanoripple surface; this is attributed to the formation of micro/nano-layered patterns and the result is improved capillary action. Pardal et al. [18] performed spot welding on steel after forming a surface microstructure. Molten aluminum deeply penetrated the steel surface microstructure, and the maximum tensile shear strength of the joint increased by 25%. The machined surface microstructure played a role in homogenizing the nugget of the solder joint and improved the welding quality. Zhang et al. [19] fabricated a surface microstructure to achieve brazing between Al_2_O_3_ and stainless steel. The maximum joint strength reached 66 MPa, which was 2.7 times that of the untreated brazed joint. The spacing between the surface grooves affected the joint performance. When the groove spacing was large, the stress distribution in the brazing process improved, and it was difficult for the cracks to expand.

The difference in the coefficient of thermal expansion and elastic modulus of ceramic–metal materials leads to the generation and concentration of residual stress in brazed joints. Relieving this residual stress in ceramic–metal joints is one of the main research directions. Owing to the significant difference in base materials, the stress generated during the brazing process cannot be effectively relieved, and the maximum residual stress of the joint tends to concentrate on the base metal/brazing joint side, which has a higher elastic modulus. The performance of the obtained joint can be improved by adjusting the local stress via patterning. Song et al. [20] developed a new solder composed of AgCuTi powder, mixed with Si_3_N_4_ particles, and found that the dispersion distribution of Si_3_N_4_ particles reduced the mismatch in the thermal expansion coefficient and Young’s modulus between Si_3_N_4_ and TiAl base metals. Xiong et al. [21] introduced millimeter-scale periodic grooves on the C/C surface, which changed the stress distribution at the ceramic interface and increased the joint strength by 101.3%. LIPSS fabrication is an efficient and high-degree-of-freedom method that can be used to change the surface morphology and wettability of a material.

This study creatively used the microscale rice leaf structure (MRLS) in order to improve the ceramic–metal joint strength and stress. Based on the MRLS, Si_3_N_4_ ceramic and titanium alloy were joined by brazing, and the influence of the surface microstructure on the ceramic joining was analyzed.

## 2. Experimental Details

The Si_3_N_4_ ceramic was sintered by hot pressing, and the titanium alloy used was Ti_6_Al_4_V. The base materials were cut into small pieces of 15 × 15 × 4 mm^3^ using a diamond wire cutting machine, and the machined surfaces were polished. The braze filler should not only help complete the joining of Si_3_N_4_, but also meet the properties of high-temperature resistance and low activation; therefore, AgCuTi was selected as the braze filler.

### 2.1. Laser Surface Texture

The laser texture system was mainly composed of a femtosecond laser, a two-dimensional mobile working platform, a console, and an inert gas protection device. The average laser power was 110 W, the wavelength was 1064 nm, the focal diameter was 60 μm, the adjustable frequency range was 1–1000 kHz, and the pulse width range was 10–100 fs. Before processing, the Si_3_N_4_ ceramic was fixed onto a table and placed within the range of the galvanometer. A gas path was fixed on the mobile platform, and nitrogen was used as a protective gas at a flow rate of 10 cm^3^/s.

**Figure 1:**
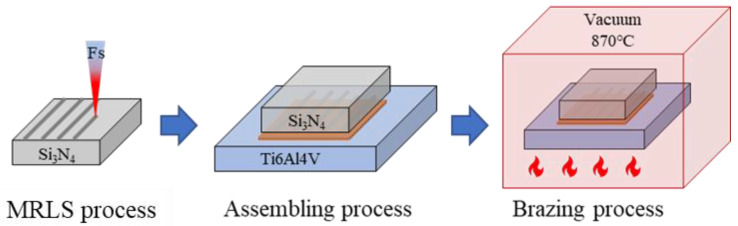
Schematics of MRLS fabrication and brazing process.

Figure 1 shows the laser scanning path. The scanning path was set along with the brazing direction, and the path was set to reciprocating to improve the processing efficiency. The distance between two adjacent paths was Δ*d*. The optimal processing parameters were as follows: line spacings of 0.01 mm and 0.1 mm, frequency of 50 kHz, scan speed of 50 mm/s, pulse width of 50 fs, and duty cycle of 50%. The roughness was measured by an optical scanning 3D profiler (Sensofar S neox).

### 2.2. Brazing and Performance Characterization

The samples to be brazed were assembled as shown in Figure 1, and then placed in a vacuum furnace. The operation program was set according to the brazing curve, and the temperature was increased to 800 °C at a rate of 10 °C/min and held for 5 min. The samples were then heated to a brazing temperature of 870 °C at a rate of 10 °C/min, held for 10 min, and finally cooled in a furnace. During the brazing process, the vacuum degree was maintained at 1~7 × 10^−3^ Pa. The shear strength was used to evaluate the mechanical properties of the joints.

## 3. Results and Discussion

### 3.1. Laser-Textured Surface Microstructure

**Figure 2:**
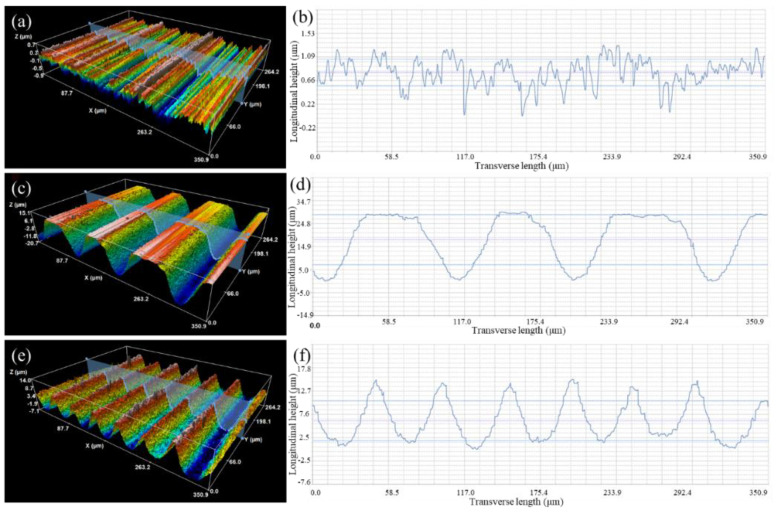
Surface profile shape curve of silicon nitride: (**a**,**b**) pristine surface; (**c**,**d**) loose surface; and (**e**,**f**) dense surface.

Figure 2 shows the three-dimensional morphology and roughness of the Si_3_N_4_ ceramic surface texture. The MRLS was obtained at a laser power of 110 W; line spacings of 100 μm (loose type) and 50 μm (dense type); and the corresponding trench structures were loose and dense, respectively.

**Figure 3:**
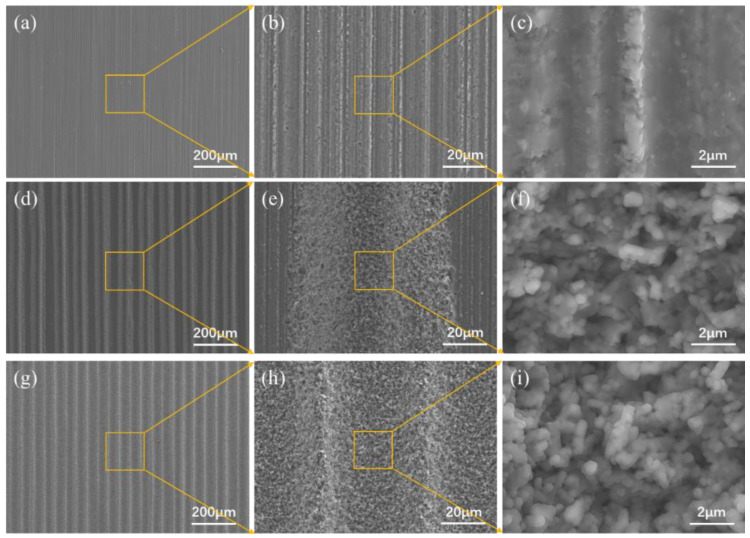
MRLS microstructure of Si_3_N_4_: (**a**) pristine surface; (**b**) groove of pristine surface; (**c**) microstructure and roughness of pristine surface; (**d**) loose surface; (**e**) high magnification of loose surface after laser processing; (**f**) nanoparticles of loose surface; (**g**) dense surface; (**h**) high magnification of dense surface after laser processing; and (**i**) nanoparticles of loose surface.

The surface of the pristine Si_3_N_4_ was relatively flat and had evident stripes with a small roughness (0.18 μm, as shown in Figure 2a). Figure 2b shows the loose groove-like texture on the surface after laser processing, with a line spacing of 100 μm. The grooves were large, the depth reached 35.79 μm, and the roughness increased to 9.83 μm. During laser texturing, the line spacing was larger than the width of a single ridge, and the micro–nano structures, formed on the previous path, were not etched in the next round of laser scanning. Figure 2c shows the formation of a dense, groove-like, textured surface with parallel, ridge-like protrusions, and the width of a single protrusion was approximately 100 μm. The center line of the ridge-like protrusion coincided with the scanning path of the laser, in which the tiny grooves and larger grooves constituted a “light trap” structure. During laser texturing, the Si_3_N_4_ surface quickly absorbed heat and melted, and small holes with large aspect ratios were thus formed. The pressure generated by the steam in the small holes squeezed the vaporized Si_3_N_4_ to move to both sides and form ridged protrusions after solidification (Figure 3f,i). The roughness was 3.71 μm, and the groove depth reached 21.11 μm.

As can be seen from the pristine Si_3_N_4_ shown in Figure 3a–c, its surface is relatively flat, with evident stripes, and small surface roughness; further, Figure 3b shows the microscopic topography formed on the loose groove-like surface. Laser-ablated Si_3_N_4_ has an affected width of approximately 40 μm. Figure 3c shows the profile curve of a single ridge and a small groove in the middle texture. The entire curve was regular, exhibiting a “V” shape. The height of the two upward growing columnar protrusions was 21.11 μm, and the gap was approximately 60 μm. The tiny grooves and larger width grooves constituted a light trap structure.

Groove structures of two different sizes were observed using SEM; Figure 3 shows the results. Owing to the induction mechanism (of ultra-short pulses) of the femtosecond laser, numerous nano-substructures, covering the entire surface of the microgrooves, appeared on the surface of the microscale grooves [22]. The nano-substructures were nanoparticles with sizes ranging from tens to hundreds of nanometers, exhibiting a coral-like structure after agglomeration. In the following brazing work, these nanoparticles were expected to provide a large number of nucleation sites for the solidification of the liquid solder, due to its high surface activity [23]. Notably, the nanoparticle cluster structure showed porous characteristics, which further improved the surface roughness of the hierarchical structure. In addition, the grooves were not very homogeneous, but the uniformity of the texture did not have an obvious effect on the wetting.

### 3.2. Performance Improvement of Brazed Joints by Textured Surfaces

**Figure 4:**
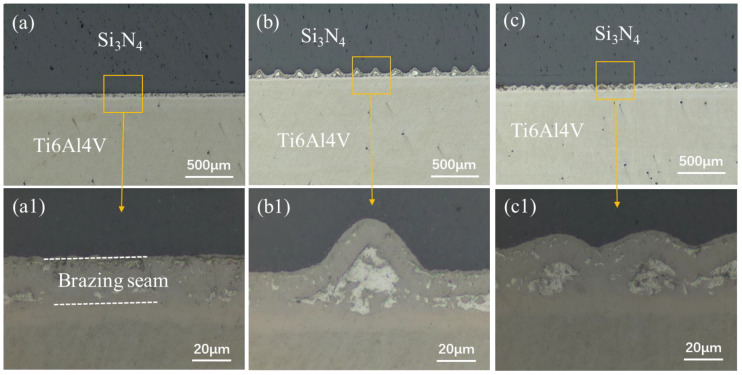
Interfacial microstructure of brazed joints of Si_3_N_4_–titanium alloys with different surface structures: (**a**,**a1**) pristine surface; (**b**,**b1**) loose surface; and (**c**,**c1**) dense surface.

**Figure 5:**
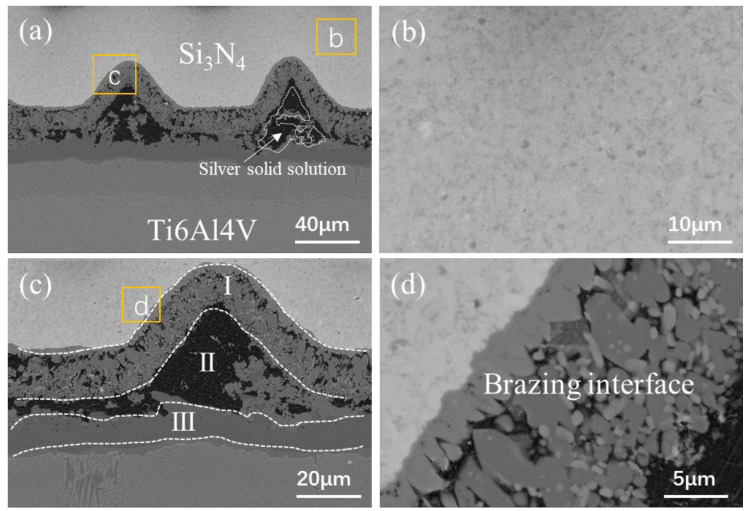
Brazing interface of the Si_3_N_4_–titanium alloy joint with a loose surface: (**a**) low-magnification microstructure; (**b**) the microstructure of Si_3_N_4_; (**c**) low-magnification microstructure (the black area shows holes that are not filled, which should contain the silver solid solution); and (**d**) brazing interface.

**Figure 6:**
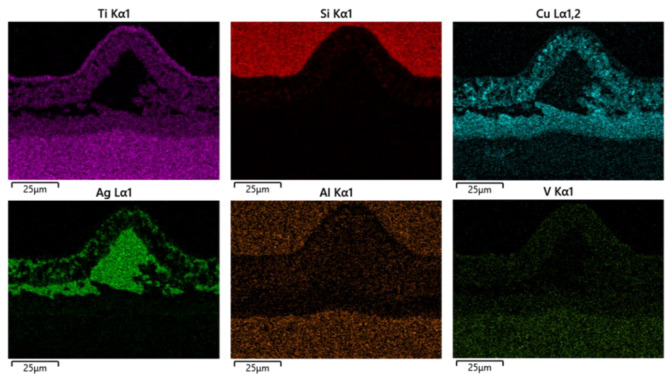
Elemental mapping of Si_3_N_4_–titanium alloy joint with a loose surface.

The MRLS was designed to improve the mechanical properties of Si_3_N_4_–titanium alloy joints and to reduce the joining defects. Figure 4 shows the typical interface structure of the joint obtained by brazing Si_3_N_4_ under a brazing temperature of 870 °C and a holding time of 10 min as the process parameters, and using MRLS to assist the AgCuTi braze filler. After brazing, there were no hole defects at the brazing interface, and the formation was good. Based on the microstructural distribution characteristics of the brazing seam interface, the interface of the brazed Si_3_N_4_–titanium alloy joint was divided into three regions: I, II, and III, and the overall width of the brazing seam was approximately 25 μm, as shown in Figure 5. Regions I and III have the same organization, which is the interfacial reaction region near Si_3_N_4_ and the titanium alloy; further, region II is the center region of the brazing seam. Throughout the brazed joint, the MRLS remained intact and formed strong metallurgical bonds with the brazing filler metal. An enlarged view of Region I shows that the central region of the brazing joint contains a gray matrix and white dotted phases; additionally, the gray phases were tightly connected to Si_3_N_4_ and facilitated by nanoparticles. Based on the elemental analysis (Figure 6), the white phase is Ti–Cu, and the white dotted phase is the silver solid solution.

**Figure 7:**
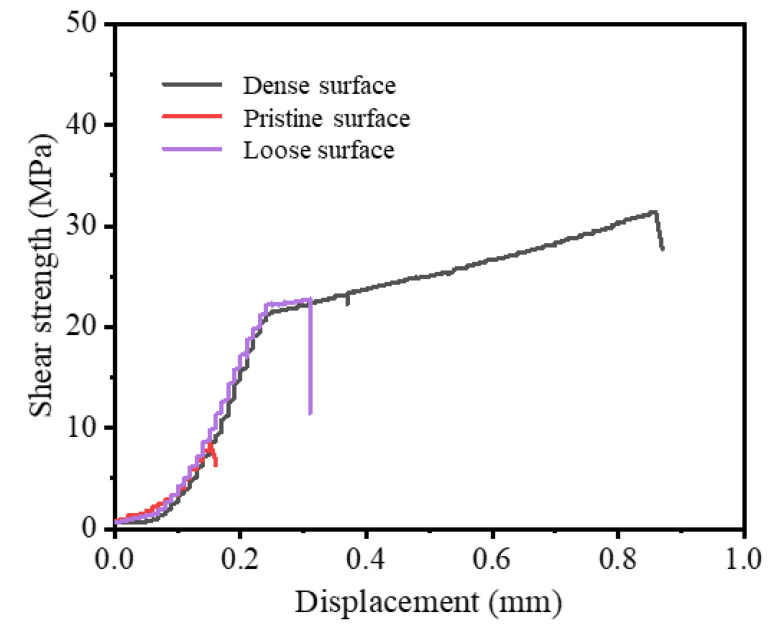
Force–displacement curve of the brazed joint.

Figure 7 shows the force–displacement curves of the Si_3_N_4_–titanium alloy joints before and after the introduction of MRLS. The holding time was 10 min, the brazing temperature was 870 °C, and the room-temperature shear strength of the joint increased gradually. The maximum shear strength was only 10 MPa without the MRLS, and the joint strength improved significantly after the introduction of MRLS. When a dense structure was adopted, the strength increased by more than 300%. Interestingly, the displacement of the joint increased significantly during the shearing process with the introduction of the MRLS, indicating a considerable improvement in the impact resistance of the joint. Based on the texture characteristics of the MRLS, we believed that the large increase in shear displacement was attributed to the groove structure, which enhanced the mechanical interlocking ability between the brazing seam and Si_3_N_4_.

**Figure 8:**
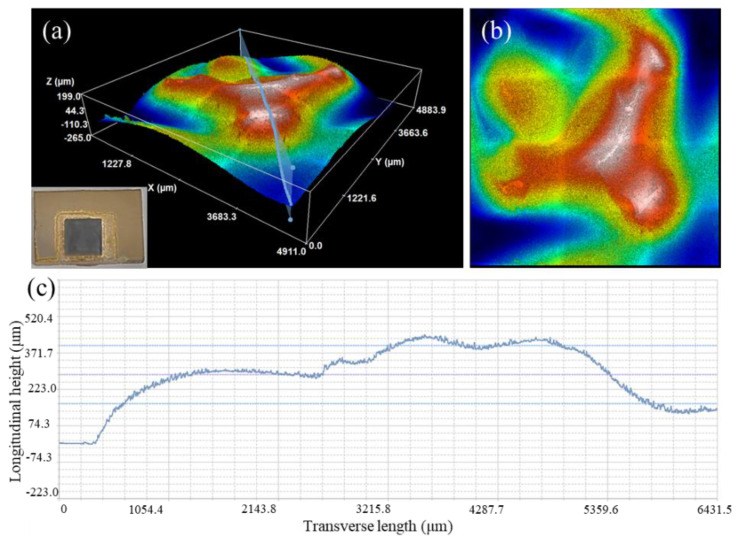
Fracture morphology of the joint after direct brazing. (**a**) three-dimensional view of the fracture morphology, (**b**) top-view of the fracture morphology, (**c**) height profile on the cross-section in figure (**a**).

The above analysis showed that the shear strength of the brazed joints significantly improved after the introduction of the MRLS. When the AgCuTi filler was used for direct brazing, the joint broke on the Si_3_N_4_ side, indicating a high residual stress on this side after direct brazing, as shown in Figure 8. This was due to the significant differences in the thermal expansion coefficient between Si_3_N_4_ and the filler metal. At the same time, the shear strength of Si_3_N_4_ was low; therefore, it was damaged along the weak area under the action of an external force, resulting in a low shear strength of the joint.

**Figure 9:**
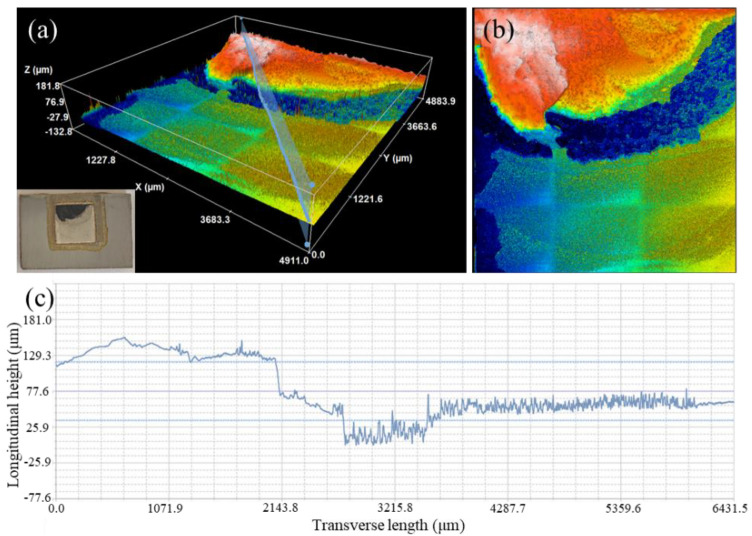
Fracture morphologies of brazed joints after introducing the MRLS. (**a**) three-dimensional view of the fracture morphology, (**b**) top-view of the fracture morphology, (**c**) height profile on the cross-section in figure (**a**).

After the introduction of the MRLS, a small amount of AgCuTi remained on the fracture surface of the joint, indicating that a part of the brazed joint area was also fractured, as shown in Figure 9. The fracture path of the joint, after brazing with the MRLS, had changed significantly. The initial fracture position was still the edge position of Si_3_N_4_, near the interface. However, the crack no longer propagated only along the Si_3_N_4_ interior; instead, it passed through the interface reaction layer and the brazing seam area.

### 3.3. Simulation Analysis of Joint Residual Stress

To further verify the effect of the MRLS on the joint, the residual stress of the joint was simulated using ABAQUS software. The strengthening mechanisms of the joint were revealed by comparing the magnitude and distribution of the joint residual stress with and without the MRLS.

**Figure 10:**
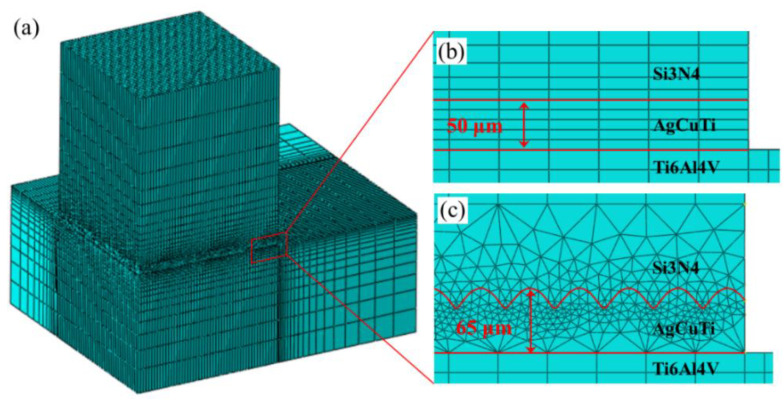
(**a**) Si_3_N_4_–titanium alloy joint model, (**b**) pristine surface, and (**c**) dense surface of the brazed joint.

A finite element analysis (FEA) was performed according to the theory of thermoelasticity and plasticity. ABAQUS software was used to simulate the residual stress field of the brazed joint during cooling and the temperature was reduced from 870 °C to 20 °C. Since the static implicit analysis step was used to solve the problem, the simulation time had no effect on the simulation results. The grid division module used C3D8R cell type. The original sample had 133,740 cells and 143,027 nodes; the dense groove joint sample had 361,176 units and 472,474 nodes. It has been assumed that only elastic deformation occurred in Si_3_N_4_, and elastic–plastic deformation occurred in the brazing layer. The various performance parameters of materials required for the finite element calculation can be found in the literature [24,25,26]. To directly relate the residual stress to the shear strength, a finite element mesh was drawn with the size of the actual shear specimen. The sizes of the Si_3_N_4_ and titanium alloy were 5 × 5 × 3 mm^3^ and 15 × 10 × 3 mm^3^, respectively. In consideration of the distribution characteristics of the residual stress of the brazed joints, a nonuniform mesh division of the brazed joints was performed. The meshes in the area near the interface, between the brazed joint and the base metal, were relatively dense, which therefore improved the accuracy of the simulation results. Figure 10 shows these meshes.

**Figure 11:**
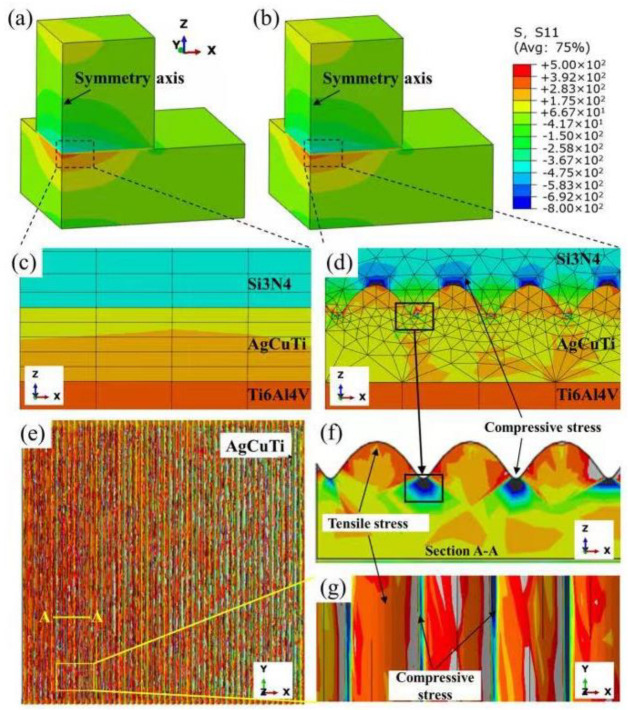
(**a**) Distribution cloud diagram of the residual stress τ_zx_ in the pristine surface, (**b**) dense surface joint, (**c**) pristine surface, (**d**) local enlarged view of the dense surface joint, (**e**) AgCuTi in the dense surface joint residual stress distribution, (**f**) corresponding local cross-section, and (**g**) surface stress state.

**Figure 12:**
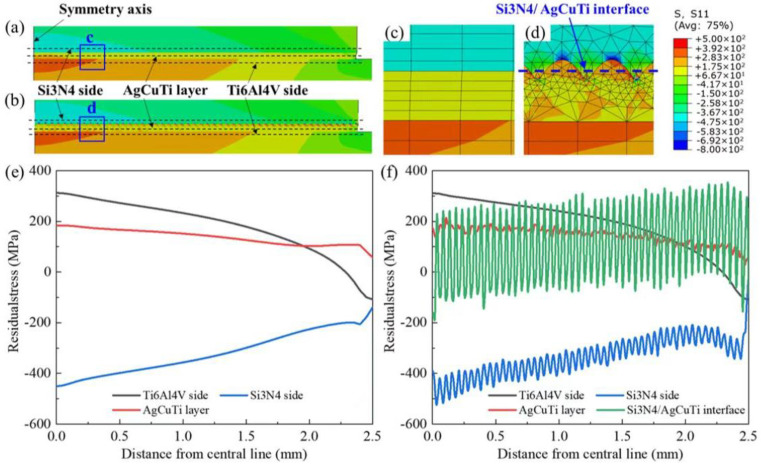
(**a**) Pristine surface, (**b**) residual stress τ_zx_ results of the dense surface joint and the schematic of the extraction position, (**c**,**d**) partially enlarged views, (**e**) pristine surface, and (**f**) residual stress distribution curve of the dense surface joint.

The initial condition considered for the finite element simulation was that the filler was a liquid before the temperature dropped to the solidification temperature of the brazing joint. Further, the residual thermal stress generated at this stage was not considered. Figure 11 shows the von Mises equivalent residual stress distribution of the brazed joint, and the corresponding residual stress distribution curve is shown in Figure 12.

For the joints directly brazed with AgCuTi, an evident stress concentration formed in the brazing seam, as shown in Figure 11 and Figure 12. There was also a stress concentration phenomenon at the corners of Si_3_N_4_; however, it was much lower than that of the brazing joint. These two locations in the joint are prone to the initiation and propagation of cracks. The internal stresses of the upper Si_3_N_4_ and lower titanium alloy were symmetrically distributed; however, the residual stress value of Si_3_N_4_ near the seam was higher. Therefore, it is more likely to break from Si_3_N_4_ when close to the brazing seam.

After the introduction of the MRLS, the stress of the joint did not increase; instead, a stress transition region was formed, which avoided any instantaneous change in stress and improved the toughness of the joint. In addition, although it was judged that the residual stress had not significantly improved on the whole, it was proven that the introduction of the MRLS structure did not damage the joint; therefore, the advantages of the MRLS structure outweigh the disadvantages.

The ceramic side of the joint was subjected to tensile and shear stresses that were directed toward the center, and the joint was also subjected to compressive and shear stresses. Si_3_N_4_ is prone to cracks under the composite state of tensile and shear stress. Therefore, cracks initiated near the brazing seam. During the crack propagation process, it was noted that the compressive stress hindered crack propagation. Combining the finite element analysis, cross-sectional topography after joint fracture, and load–time curve, it was found that the MRLS not only improved the mechanical and brazing area at the interface, but also produced a compressive stress on the ceramic side. This hindered the expansion of cracks, thereby playing a significant role in improving the joint strength.

## 4. Discussion

The MRLS design strategy was implemented in brazing in order to significantly increase joint strength. In this work, the groove array structure, which was fabricated by a femtosecond laser, was uniformly distributed on the Si_3_N_4_ surface, resulting in a significantly increased surface area. Under the action of laser ablation, a large number of nanoparticles were fully covered on the groove surface (see Figure 3). During brazing, these nanoparticles provide nucleation points for the solidification of the liquid solder, resulting in a dense interface connection (see Figure 5). This excellent interface connection, combined with mechanical interlocking, significantly improved the shear resistance of the joint. Residual stress was an unavoidable problem in brazing the connection, as this was derived from the huge physical differences (such as the linear expansion coefficient and elastic modulus), between the ceramics and metals. In severe cases, the ceramic side directly broke due to excessive internal stress (Figure 8). The introduction of a groove structure created a stress transition zone between the metal and ceramic. This, therefore, reduced the risk of direct fracture in the ceramic, thereby shifting the load capacity from brittle ceramics to the high-quality brazing seams (Figure 9). The finite element analysis showed that the periodicity of compressive stress along the interface further inhibited crack propagation. In summary, the MRLS induced a composite effect of dense interface, mechanical occlusion, and stress transition, which, taken together, promoted the bonding strength of the brazed joints.

## 5. Conclusions

(1) The MRLS was obtained at a laser power of 110 W, with line spacings of 100 and 50 μm. A number of nanoparticles was formed during laser processing, with particle sizes ranging from tens to hundreds of nanometers, exhibiting a coral-like structure after agglomeration.
(2) After brazing with the MRLS, there were no defects at the brazing interface, and the formation was good. The interface of the brazed Si_3_N_4_–titanium alloy joint was divided into three regions, and the central zone of the joint contained a gray Ti–Cu matrix, and a silver solid solution.
(3) The joint strength was significantly improved with the implementation of the MRLS, and the displacement of the joints increased significantly during the shearing process. After brazing with the MRLS, the fracture path changed significantly, and the maximum shear strength reached about 35 MPa. The periodic microgrooves on the surface not only improved the mechanical occlusion and brazing area at the interface, but also generated compressive stress on the Si_3_N_4_ side.

## Data Availability

Data will be made available on request.
